# A novel type V natural deep eutectic solvent with built-in pH-regulation for magnetic dispersive liquid-liquid microextraction of central nervous system drugs in plasma samples

**DOI:** 10.3389/fchem.2026.1986305

**Published:** 2026-10-27

**Authors:** Wen-Chao Han, Yu-Ling Yang, Xiao-Bei Yang, Han-Juan Zhang, Yong-Yan Chen, Xi-Rui Pei, Dong-Dong Yuan, Di Chen

**Affiliations:** 1 Department of Pharmacy, The Seventh People’s Hospital of Zhengzhou, Zhengzhou, China; 2 Institute of Biological Therapy, Henan Academy of Innovations in Medical Science, Zhengzhou, China; 3 Clinical Trial Institution for Drugs of Pharmacy Department, The Second Affiliated Hospital of Zhengzhou University, Zhengzhou, China; 4 School of Pharmaceutical Sciences, Zhengzhou University, Zhengzhou, China; 5 Children’s Hospital Affiliated to Zhengzhou University, Zhengzhou University, Zhengzhou, China

**Keywords:** alkaline natural deep eutectic solvent, central nervous system drugs, gas chromatography-mass spectrometry, green sample preparation, magnetic dispersive liquid-liquid microextraction, plasma

## Abstract

**Introduction:**

This study proposes an alkaline natural deep eutectic solvent (ANADES)-based magnetic dispersive liquid-liquid microextraction (ANADES-M-DLLME) method for the convenient, green, and efficient extraction of CNS drugs from plasma.

**Methods:**

A novel, non-toxic type V ANADES, serving as the extractant, not only allows for polarity customization, but also directly adjusts the solution’s pH to alkaline conditions due to its built-in pH regulation, significantly improving the extraction efficiency of CNS drugs. Moreover, the elimination of the external pH adjustment, combined with magnetic particle-assisted retrieval of the extractant, substantially streamlines the sample preparation process.

**Results:**

Under the optimal extraction conditions, the developed method, coupled with GC-MS/MS detection, was successfully applied for the extraction and quantification of CNS drugs in human plasma samples. The validated method exhibited good linearity (*R* ^2^ > 0.990), with high accuracy (spiked recoveries 95.1%–114.9%) and precision (RSDs < 13.3%). Low limits of detection (0.61–3.36 ng/mL) and quantification (2.05–9.89 ng/mL), satisfactory extraction recoveries (85.01%–99.89%), and enrichment factors (6.65–8.79) were achieved for the seven target analytes. The analysis of the proposed method using three green analytical chemistry metrics further substantiated the method's greenness.

**Discussion:**

Compared with reported DLLME techniques, the established ANADES-M-DLLME method was green, rapid, convenient, and cost-effective, offering a promising solution for widespread clinical and bioanalytical applications.

## Graphical Abstract

**Figure:**
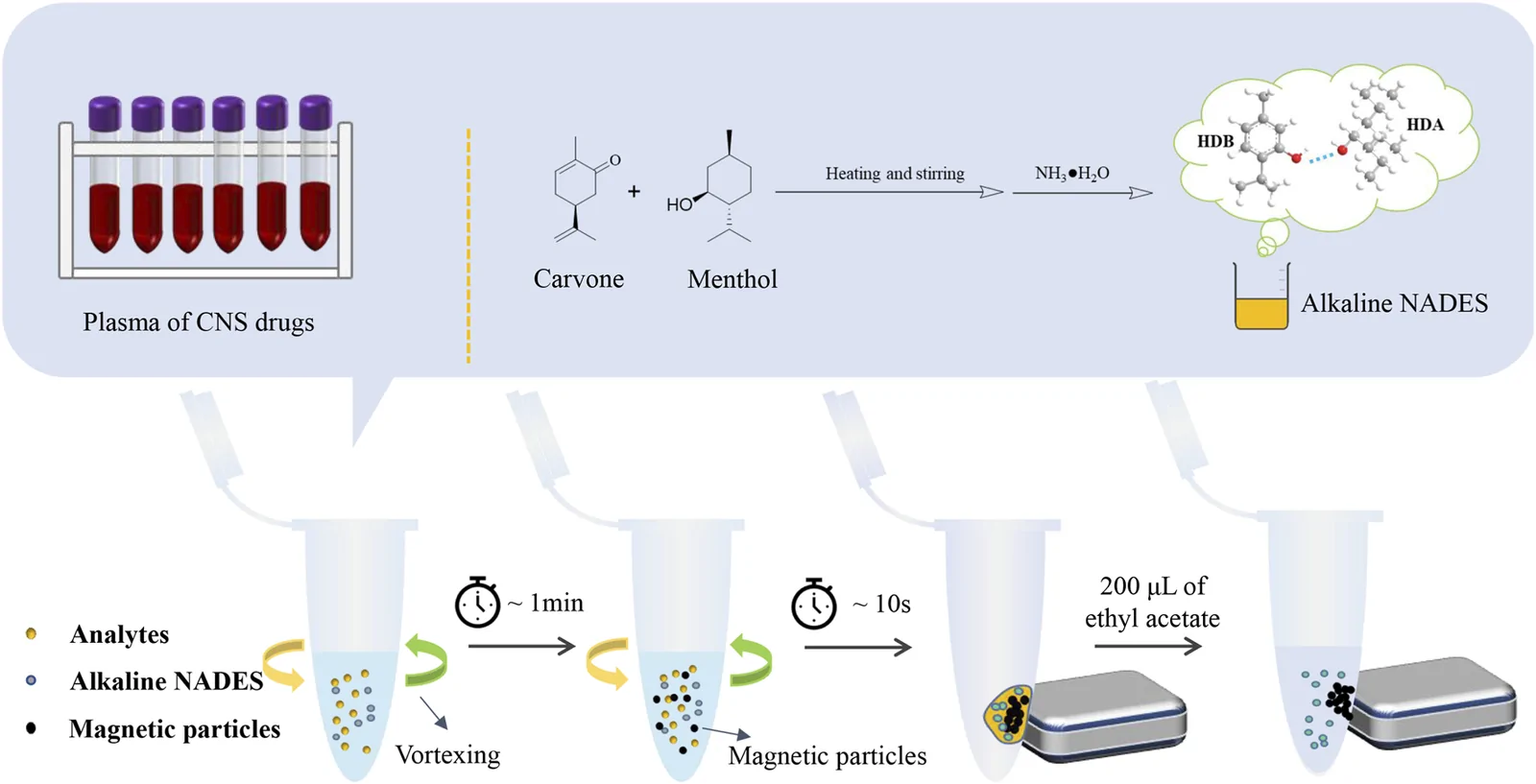

## 1 Introduction

Accurate monitoring of central nervous system (CNS) drugs in biological fluids is critical for clinical toxicology and therapeutic drug management (Pennazio et al., 2022). These CNS drugs, such as antidepressants, sedative-hypnotics, and antipsychotics, commonly possess narrow therapeutic windows, necessitating precise blood concentration measurements to prevent complications such as overdose, toxicity, and therapeutic non-response (An et al., 2021; Moschny et al., 2021; Reis et al., 2009). However, chromatographic analysis of biological samples presents significant challenges owing to matrix complexity, particularly from plasma proteins and phospholipids (Deng et al., 2025). Consequently, effective sample preparation is essential prior to chromatographic instrument analysis.

To date, miniaturized extraction techniques, offering advantages including minimal solvent consumption, low cost, and high efficiency, have been widely employed. Examples include magnetic solid-phase extraction (MSPE) (Wang et al., 2025; Zhang et al., 2024), hollow fiber-based solid-phase microextraction (HF-SPME) (Li et al., 2020), and microextraction by packed sorbent (MEPS) (de Souza et al., 2015; Soares et al., 2024). Dispersive liquid-liquid microextraction (DLLME) represents one of the most commonly used miniaturized forms, providing a simple extraction process and high enrichment in different sample matrices (Notardonato and Avino, 2024; Sajid and Alhooshani, 2018). Within DLLME, the selection of the extraction solvent is particularly critical. However, the varying polarities of analytes limit the improvement of extraction efficiency when using a single solvent for multi-analyte extraction. Binary-solvent DLLME (BS-DLLME) (Dalgıç Bozyiğit et al., 2021), utilizing two complementary extraction solvents, partially mitigates the limitations of conventional single-solvent DLLME for multi-analyte extraction. There remains, however, a persistent drawback for these techniques: heavy reliance upon volatile, toxic organic solvents as extractants, posing severe toxic effects on humans and the environment. Given the principles of green analytical chemistry, developing non-toxic, efficient, and low-cost green extractants has become a hotspot in many DLLME technique studies (Grau et al., 2022; Kokosa, 2019). In this context, deep eutectic solvents (DESs) have attracted much attention (El-Deen and Shimizu, 2021). DESs are multi-component solvents formed by mixing a hydrogen bond acceptor (HBA) and a hydrogen bond donor (HBD) via hydrogen bonding interactions. The integration of DESs with DLLME is increasingly recognized as a viable alternative to traditional organic reagents (Musarurwa and Tavengwa, 2021; Rageh et al., 2024). Compared to conventional solvents, DESs are not only facile to synthesize but their unique composition allows polarity customization. This tunability enables the extraction of a broad spectrum of hydrophobic and hydrophilic chemicals, facilitating efficient multi-analyte extraction from both aqueous and oily samples (Kodalak and Bişgin, 2026). In recent years, natural deep eutectic solvents (NADES), composed of hydrophobic natural compounds (such as menthol, L-lactic acid, fatty acids, etc.), have been increasingly developed (Bişgin et al., 2025; Cherniakova et al., 2024; Florindo et al., 2018). Notably, the introduction of type V DES (composed of non-ionic HBA and HBD) has further expanded the selection range of NADES (Schaeffer et al., 2021). These solvents combine advantages such as non-toxicity, cost-effectiveness, and biodegradability, making them highly attractive options for green analytical chemistry (Shishov et al., 2024).

Although NADES-based DLLME methods have been reported, they generally suffer from two limitations. On one hand, when extracting amphoteric compounds, pH adjustment is commonly essential to enhance extraction selectivity and efficiency. Existing methods often necessitate supplementary steps, such as pH adjustment using buffers, acids, or bases, to achieve optimal extraction conditions for different analytes (Fernández et al., 2016; Qiao et al., 2021; Yıldırım et al., 2024). This imposes additional operational complexity and time requirements while potentially introducing analytical errors, thereby compromising method robustness and reproducibility. Although the use of NADES in analytical chemistry, including DLLME, is expanding, reports on their combination with pH-regulation techniques are scarce (Majidi and Hadjmohammadi, 2020; Yang et al., 2019). To the best of our knowledge, only a few acidic DESs have been reported for analytical-scale extraction, in which acidity generally originates from acidic hydrogen-bond donors constituting the DES (Zhu et al., 2021). In contrast, alkaline DESs prepared in a similar manner and applied for analytical-scale extraction have not been reported. In particular, a type V alkaline NADES with built-in pH regulation achieved by incorporating base into a hydrophobic NADES has not been described. This represents a distinct strategy compared with conventional external pH adjustment using buffers, acids, or bases. On the other hand, convenient retrieval of microvolumes of extractant remains a challenge in DLLME, irrespective of whether DESs or traditional solvents are employed. Efforts have recently been made in DLLME, such as in-syringe set-up or solidification of floating organic droplets (SFOD) (Jing et al., 2019; Maya et al., 2014), however, the results remain unsatisfactory, and the extraction process is still relatively complex and labor-intensive. Given these considerations, developing a more environmentally friendly, streamlined, and efficient DLLME approach is imperative.

This study aims to develop a novel sample pretreatment approach based on type V alkaline natural deep eutectic solvent-magnetic dispersive liquid-liquid microextraction (ANADES-M-DLLME) for the simple and efficient extraction of CNS drugs from biological samples. Unlike previously reported NADES-based DLLME methods, the present work introduces a novel type V alkaline NADES (ANADES) with built-in pH regulation, prepared by incorporating ammonia into a hydrophobic NADES. This design eliminates external pH adjustment. Moreover, magnetic Fe_3_O_4_ particles are used to retrieve the NADES phase, enabling rapid and convenient phase separation. To the best of our knowledge, this is the first report of a type V alkaline NADES combined with magnetic dispersive liquid–liquid microextraction (ANADES-M-DLLME) for the extraction of basic CNS drugs from plasma. The proposed method is therefore clearly distinguished from previously reported NADES-based DLLME approaches in terms of pH regulation strategy, extractant retrieval, and operational simplicity.

## 2 Experimental section

### 2.1 Reagents and materials

Unless otherwise specified, analytical grade chemicals and reagents were used throughout this study. Pharmaceutical standards, including lidocaine (LID, ≥98%), chlorpromazine (CMZ, ≥99.81%), clozapine (CZP, ≥99%), and the internal standard (IS) diphenylamine (DPM, ≥98%), cyclobenzaprine hydrochloride (CBZ, ≥98%), were purchased from damas-beta Co., Ltd. (Shanghai, China). Amitriptyline (AMP, ≥98%) and promethazine (PMZ, ≥99%) were supplied by Aladdin Reagent Co., Ltd. (Shanghai, China), while imipramine hydrochloride (IMP, ≥99.93%) was obtained from Bide Pharmatech Ltd. (Shanghai, China). Diazepam (DZP, 2.5 mg/tablet) was purchased from Huazhong Pharmaceutical Co., Ltd. (Wuhan, China).

DL-Menthol (≥98%) and (R)-(-)-carvone (≥99%) were purchased from damas-beta Co., Ltd., and eucalyptol (≥99%) was obtained from Ron Reagent Co., Ltd. (Shanghai, China), all serving as components for the synthesis of NADES. Solvents involved in the experiment—ethyl acetate (analytical grade), ethanol (analytical grade), and acetonitrile (HPLC grade)—were purchased from Tianjin Fengchuan Chemical Reagent Technology Co., Ltd. (Tianjin, China), with additional HPLC-grade ethyl acetate supplied by Chemicell Co., Ltd. (Berlin, Germany). Magnetic (Fe_3_O_4_) particles (20, 50, and 500 nm) were provided by Shanghai Zhongye New Materials Co., Ltd. (Shanghai, China).

### 2.2 Apparatus

Sample homogenization was achieved using a multi-tube vortex mixer (BTV-100, Yiheng, Shanghai, China). Separation operations were conducted with a high-speed centrifuge (TG18.5, Luxiangyi, Shanghai, China), while micro-scale centrifugation employed a mini palm centrifuge (LC-Mini-4K, Lichenbangxi, Shanghai, China). Solution pH adjustments were performed with a calibrated pH meter (PHS-3E, Yidian, Shanghai, China). A heated magnetic stirrer (LC-OB-2L, Lichenbangxi, Shanghai, China) facilitated temperature-controlled synthesis reactions.

### 2.3 Preparation of solutions

Stock solutions (1.0 mg/mL) of the seven CNS drugs were prepared in methanol and stored at 4 °C. Working solutions containing all seven analytes were freshly diluted with deionized water to the target concentrations prior to use. Plasma samples, obtained from the Seventh People’s Hospital of Zhengzhou, were stored at −80 °C in the dark prior to analysis. Plasma samples were allowed to thaw to room temperature (∼25 °C) before use. Spiked plasma samples were prepared by accurately adding a specific volume of a mixed stock solution to blank biofluid, followed by dilution to the desired concentrations. For analysis, 300 μL of plasma was combined with 700 μL of ethanol. The mixture was centrifuged, and the resulting supernatant was diluted 1:2 (v/v) with deionized water. The final dilution factor (6.33) of the plasma sample was calculated: Dilution factor = final dilution volume/original plasma volume. This diluted supernatant was then subjected to the proposed alkaline natural deep eutectic solvent-based magnetic dispersive liquid-liquid microextraction (ANADES-M-DLLME) procedure.

### 2.4 NADES preparation

A series of novel type V NADES were synthesized using a heating-stirring method (Zamora et al., 2022). Menthol served as the hydrogen bond donor (HBD), while eucalyptol or carvone acted as the hydrogen bond acceptor (HBA). Specifically, NADES 1 was prepared from menthol and eucalyptol, and NADES 2 from menthol and carvone. For each NADES, stoichiometric amounts of the components (menthol paired with either eucalyptol or carvone) were mixed at varying molar ratios. The mixtures were heated in a water bath at 40 °C under continuous stirring until liquefaction. Subsequently, the molten mixtures were vortexed for 1 min to yield homogeneous transparent liquids.

### 2.5 Alkaline NADES preparation and pH validation

Alkaline NADES was prepared by vortexing 1 mL of NADES with 400 µL of ammonia (25%, w/w) for 2 min, followed by centrifugation (12,000 rpm, 2 min) and collection of the supernatant. For pH validation, 100 µL of the prepared alkaline NADES was diluted into 5 mL of deionized water and vortexed for 1 min. Subsequently, 50 mg of magnetic particles (Fe_3_O_4_, 50 nm diameter) were added. Upon vortexing (1 min), the NADES associated with these particles. The NADES-bound particles were then magnetically separated to the bottom of the tube. Finally, the pH of the supernatant was measured using a pH meter.

### 2.6 The ANADES-M-DLLME procedure

The ANADES-M-DLLME procedure is as follows, with the workflow illustrated in Figure 1. Briefly, 23 μL of the alkaline NADES was added to 2 mL of pre-treated plasma sample containing CNS drugs and ISs at 50 ng/mL. The mixture was vortexed for 2 min. Subsequently, 12 mg of magnetic particles (Fe_3_O_4_, 50 nm) was introduced, followed by mixing for 30 s to form a NADES-coated magnetic composite. This composite was magnetically retrieved to the bottom of the centrifuge tube using an external magnetic field, and then the supernatant was discarded. For analyte desorption, 200 μL of ethyl acetate was added and the mixture was vortexed for 1 min. Following magnetic separation, the supernatant was transferred into a sample vial for GC-MS/MS analysis.

**FIGURE 1:**
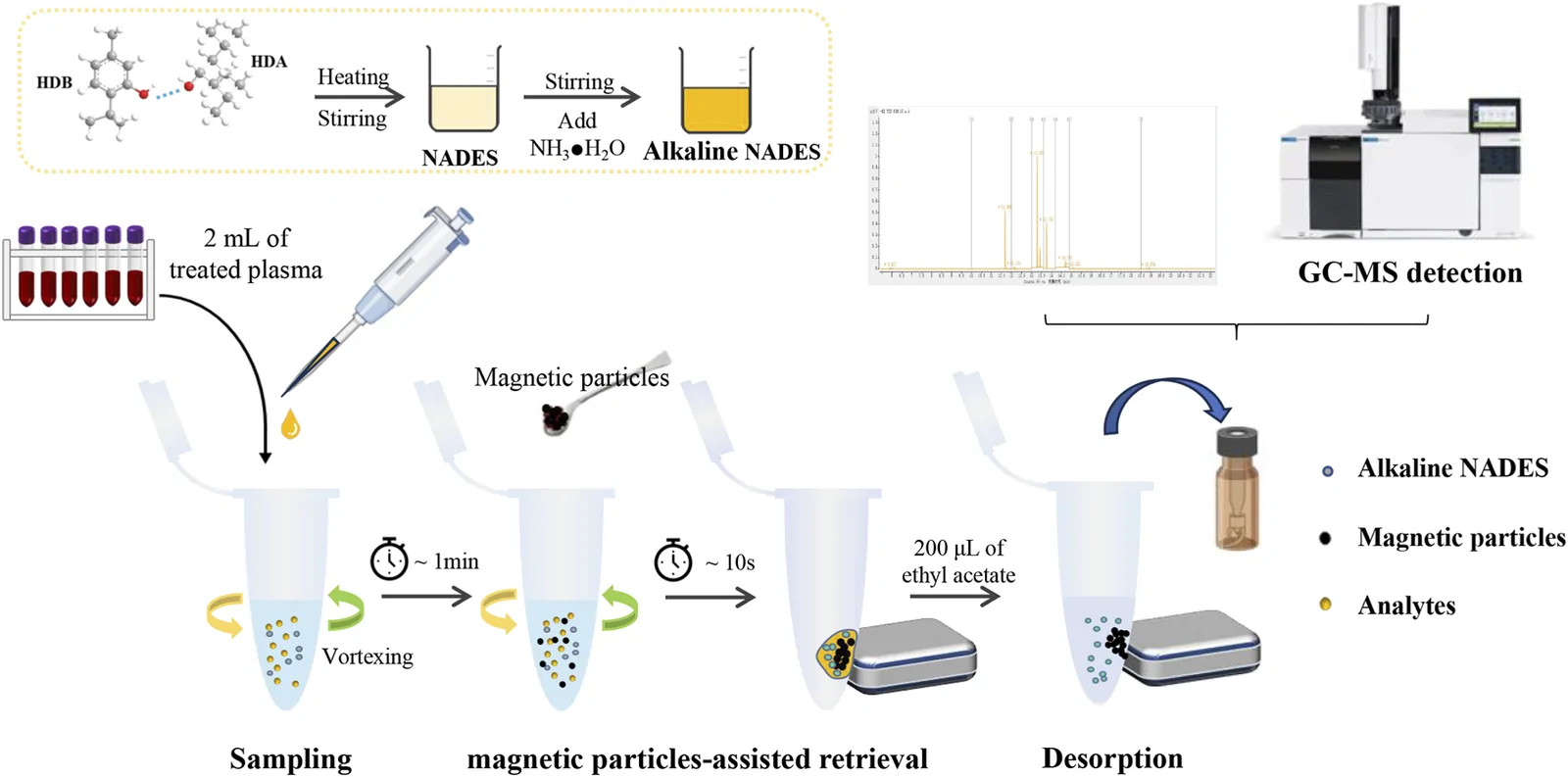
The schematic diagram of the proposed ANADES-M-DLLME method.

### 2.7 GC-MS/MS analysis

Two GC-MS systems, the Agilent 8890-5977B and 8890-7000D GC-MS/MS systems (Agilent, Santa Clara, United States), were used for the analysis of selected CNS drugs. Chromatographic separation of these two systems was achieved on an HP-5 capillary column (30 m × 0.25 mm, 0.25 μm; Agilent, Santa Clara, United States) with helium carrier gas at a constant flow rate of 1.0 mL/min. The injector temperature was maintained at 280 °C, with an injection volume of 1 μL, utilizing the splitless injection mode. The oven temperature program initiated at 80 °C (3 min hold), ramped at 20 °C/min to 280 °C (25 min hold), yielding a 38 min total runtime. Mass spectrometry conditions were as follows: electron ionization at 70 eV with ion source (260 °C) and transfer line (270 °C). For data acquisition, extraction optimization, an Agilent 8890-5977B (single quadrupole) was operated in Selected Ion Monitoring (SIM) mode. For method validation and real sample analysis, an Agilent 8890-7000D (triple quadrupole) was operated in multiple reaction monitoring (MRM) mode. Analyte-specific MRM parameters (retention times, precursor/product ion pairs, and collision energies) for the seven CNS drugs and the internal standards are detailed in Supplementary Table S1.

### 2.8 Response surface methodology (RSM) optimization

After single-factor experiments, a three-factor, three-level Box–Behnken design (BBD) was applied to optimize the key extraction parameters: volume of ANADES (X_1_, μL), mass of magnetic particles (X_2_, mg), and extraction time (X_3_, min). The average extraction recoveries (%) of seven CNS drugs were used as the response. The experimental design, data analysis, and response surface plotting were performed using Design-Expert software (version 13, Stat-Ease Inc., Minneapolis, United States). The sequence of experimental runs was randomized to minimize systematic error.

## 3 Results and discussion

### 3.1 Characterization of type V ANADES

To probe the intermolecular interactions, FTIR spectra of the individual components (menthol, carvone, and eucalyptol) and the prepared mixtures (NADES 1: menthol/eucalyptol; NADES 2: menthol/carvone) were recorded. As shown in Supplementary Figure S1, pure menthol exhibits a characteristic O–H stretching vibration peak at 3336 cm^-1^. Upon mixing menthol with eucalyptol or carvone, this O–H absorption band shifted to higher wavenumbers, appearing at 3404 cm^-1^ and 3428 cm^-1^, respectively, accompanied by noticeable changes in peak shape. Furthermore, in the fingerprint region (1500–1000 cm^-1^), the skeletal characteristic peaks of the individual components (menthol at 1450 cm^-1^, eucalyptol at 1465 cm^-1^, and carvone at 1435 cm^-1^) also exhibited slight shifts in the corresponding mixtures (e.g., appearing at 1464 cm^-1^ in NADES 1 and 1451 cm^-1^ in NADES 2). These shifts in characteristic peaks and peak broadening indicate a reorganization of the hydrogen-bonding environment and are consistent with the existence of intermolecular hydrogen-bonding interactions between menthol and the hydrogen-bond acceptors. No new covalent-bond absorption bands were observed, suggesting that the interactions are predominantly non-covalent. However, it should be emphasized that FTIR peak shifts alone cannot unambiguously confirm the formation of a NADES, because similar spectral changes may also arise from simple mixing, dilution, or disruption of the self-associated hydrogen-bonding network of menthol. Therefore, ^1^H NMR was performed as a complementary technique.

The ^1^H NMR spectroscopy was performed to investigate the intermolecular interactions (Supplementary Figure S2A and S2B). Compared with pure menthol, both DESs (NADES1 and NADES2) exhibited a distinct upfield shift (Δδ < 0) for the proton signals at approximately 2.5 ppm, while other signals remained essentially unchanged. This specific upfield shift can be attributed to the spatial anisotropic shielding effects of the rigid bicyclic structure of eucalyptol and the conjugated cyclohexenone moiety of carvone, combined with the polarization effect induced by hydrogen bonding. This consistent chemical shift variation strongly confirms the formation of intermolecular hydrogen bonds between menthol and both hydrogen bond acceptors. Taken together with the FTIR results and the formation of homogeneous transparent liquids at room temperature, these results support the formation of type V NADES.

### 3.2 Feasibility study of the ANADES-M-DLLME method

#### 3.2.1 Feasibility of ANADES synthesis

Unlike NADESs containing ionic species or acidic components such as L-lactic acid and fatty acids, type V DESs are formed solely through hydrogen-bonding interactions between neutral molecules. This non-ionic nature is particularly advantageous for constructing an alkaline NADES. If the NADES contains acidic groups (e.g., carboxylic acids in L-lactic acid or fatty acids), the addition of ammonia would trigger acid–base neutralization, consuming the alkali and disrupting the hydrogen-bonding network, thereby compromising the built-in pH regulation. In contrast, the non-ionic components of type V DESs, such as menthol, carvone, and eucalyptol, do not undergo acid–base reactions with ammonia. Ammonia can therefore be incorporated into the NADES matrix through hydrogen bonding without being neutralized, endowing the solvent with stable alkalinity. For these reasons, type V DESs were selected in this work to prepare the alkaline NADES with built-in pH regulation for the extraction of basic CNS drugs.

The feasibility of synthesizing ANADES was assessed using various alkaline compounds (sodium hydroxide, potassium hydroxide, and ammonia). Saturated solutions of these alkalis were vortexed with NADES. Successful preparation of ANADES was validated by pH measurements of sample solutions after adding NADES-alkali mixtures. Results demonstrated that only ammonia yielded functional ANADES. To provide stronger evidence for the claimed pH-regulating ability of ANADES, the pH values of pure NADES and ANADES were compared in water and pretreated plasma samples (n = 6). Pure NADES gave neutral pH in water (6.82% ± 2.98%) and in plasma (6.95% ± 3.47%). In contrast, ANADES gave clearly alkaline pH in water (9.49% ± 4.36%) and in plasma from six different donors (9.28% ± 5.27%). These results confirm that the alkalinity of ANADES originates from the incorporated ammonia and that ANADES can effectively regulate the pH of plasma samples to alkaline conditions with good reproducibility.

To further elucidate the integration mechanism of aqueous ammonia into the hydrophobic NADES, the behavior of different alkaline compounds was further investigated. After vortexing and centrifugation, the ANADES remained clear and transparent, suggesting that reverse emulsion formation is unlikely. Among the alkalis tested, only ammonia yielded a functional ANADES. This selective behavior may be attributed to the molecular nature of ammonia, which can potentially form hydrogen bonds with the NADES components (menthol hydroxyl and carvone/eucalyptol carbonyl groups). In contrast, NaOH and KOH are strong electrolytes that dissociate completely into Na^+^/K^+^ and OH^−^ ions; these ions, due to their charge and high hydration energy, cannot partition into the hydrophobic NADES phase and are removed upon centrifugation, leaving the NADES phase unchanged and devoid of alkalinity. Based on these observations, it is proposed that ammonia probably dissolves directly into the NADES matrix via hydrogen bonding, thereby endowing the ANADES with built-in pH-regulation capability without external pH adjustment. It should be emphasized that the alkalinity of the resulting ANADES originates from the incorporated ammonia, rather than from the NADES components themselves. Menthol, carvone, and eucalyptol are neutral compounds and do not possess intrinsic alkalinity; the alkaline character is imparted solely by the ammonia dissolved in the NADES matrix.

#### 3.2.2 Feasibility of the ANADES-M-DLLME process

To evaluate the extraction efficiency of the proposed ANADES-M-DLLME method for CNS drugs, comparative extractions were performed under acidic, neutral, and alkaline conditions. Following extraction, target analytes were quantified via GC-MS, with extraction recoveries calculated from their chromatographic peak areas. As illustrated in Supplementary Figure S3A and S3B, alkaline conditions significantly enhanced the extraction recoveries of all seven analytes (>80%), whereas acidic conditions gave the poorest recoveries. This pH-dependent behavior can be explained by the ionization behavior of the selected CNS drugs, which are weakly basic compounds with conjugate-acid pKa values ranging from approximately 8.0–10.5. At the optimized extraction pH of 9.49, which is above their pKa values, the analytes are predominantly present in their neutral, deprotonated forms. Under acidic conditions, they exist as protonated (ionic) species with higher water solubility and lower affinity for the hydrophobic ANADES; under alkaline conditions, they are predominantly neutral (molecular) species, favoring hydrophobic partitioning into the ANADES phase. Additionally, hydrogen bonding between the analytes and NADES components (menthol hydroxyl and carvone/eucalyptol carbonyl groups), as well as π-type interactions for aromatic drugs, may further enhance extraction efficiency. After extraction, Fe_3_O_4_ magnetic nanoparticles were added to retrieve the ANADES phase; the high recoveries (>80%) confirm that the magnetic particles efficiently captured the NADES droplets, likely facilitated by interfacial interactions such as hydrogen bonding between surface hydroxyl groups (–OH) on Fe_3_O_4_ and the polar functional groups of NADES (e.g., –OH of menthol, –C=O of carvone). Further comparison of alkaline NADES performance (Supplementary Figure S3C) revealed that alkaline NADES 2 (menthol–carvone) exhibited markedly superior extraction efficiency compared to alkaline NADES 1 (menthol–eucalyptol). Consequently, alkaline NADES 2 was selected as the optimal extraction solvent for subsequent experiments.

#### 3.2.3 Performance of the ANADES-M-DLLME method

The feasibility and performance of the newly developed ANADES-M-DLLME procedure were assessed using aqueous solutions spiked with CNS drugs at 5 μg/mL and blank aqueous solutions. Chromatographic analysis (Figure 2) demonstrated that the peaks for the spiked samples processed by ANADES-M-DLLME (curve a) corresponded well with the reference peaks of the 5 μg/mL CNS drug standard solution (curve b). Moreover, significantly enhanced signals of the analytes were observed in curve a compared to curve b, confirming the effective enrichment of the ANADES-M-DLLME method. Concurrently, the chromatogram of the blank water sample treated with ANADES-M-DLLME (curve c) exhibited no detectable peaks, indicating the absence of interference in the determination of CNS drugs. These results collectively validated the ANADES-M-DLLME procedure as a feasible approach for the enrichment of CNS drugs from aqueous samples.

**FIGURE 2:**
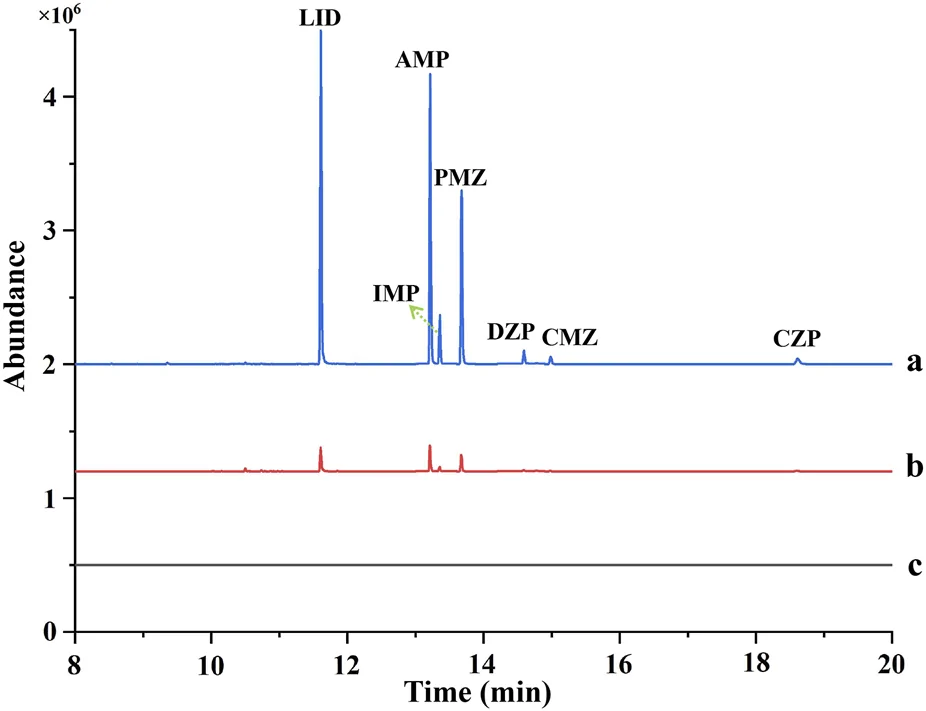
GC-MS chromatograms of **(a)** standard solution (5 μg/mL) of seven CNS drugs treated by the proposed ANADES-M-DLLME process, **(b)** standard solution (5 μg/mL) of seven CNS drugs, **(c)** the blank water sample treated by the proposed ANADES-M-DLLME process.

### 3.3 Optimization of ANADES-M-DLLME conditions

To achieve optimal extraction conditions, key parameters influencing the extraction effects of seven CNS drugs were investigated. These parameters included the HBD:HBA molar ratio, the volume of NADES, the volume of ammonia, extraction time, the mass of magnetic particles, the size of magnetic particles, desorption time, and the volume of desorption solvent. Single-factor optimization was initially employed to determine the optimal level for each parameter. Subsequently, building upon these single-factor results, critical experimental conditions were further evaluated using Response Surface Methodology (RSM). All experiments were performed in quadruplicate using standard aqueous solutions containing the analytes at 5 μg/mL. The average extraction recoveries and their standard deviations were calculated and analyzed to optimize the ANADES-M-DLLME conditions.

#### 3.3.1 Optimization of variables for the extraction conditions

The HBD:HBA molar ratio was a critical factor influencing the extraction recoveries of CNS drugs. NADES prepared from menthol and carvone at molar ratios of 1:2, 1:1.5, 1:1, 1.5:1, and 2:1 were evaluated. As shown in Figure 3A, the NADES with a 1:1 ratio yielded significantly higher recoveries compared to other ratios. This can be attributed to the optimal polarity match between the NADES and the target analytes at the equimolar ratio, which provides the most favorable partitioning environment for the CNS drugs. Consequently, this menthol-carvone NADES (1:1) was selected for further work.

**FIGURE 3:**
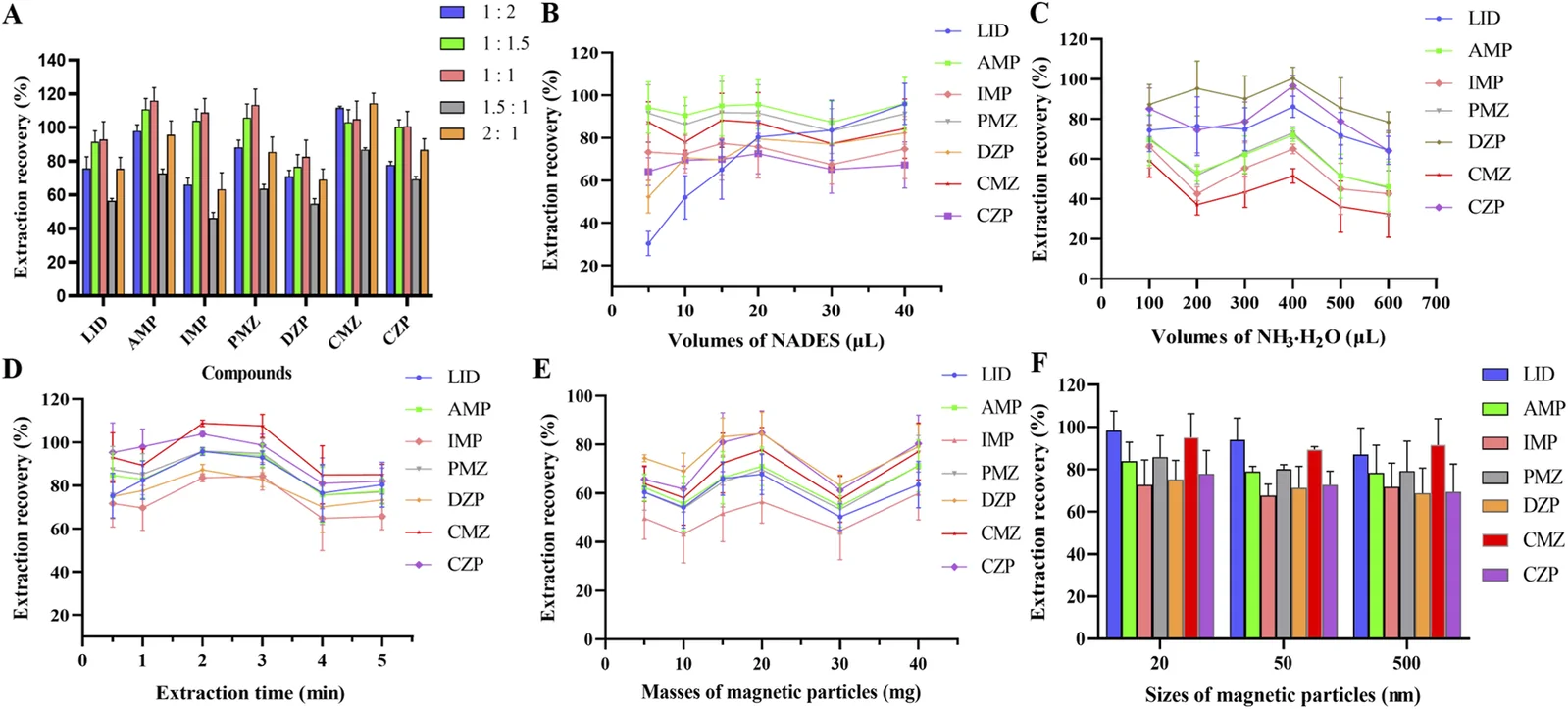
Optimization of the extraction conditions of the ANADES-M-DLLME method using single-factor experiments: **(A)** HBD:HBA molar ratio, **(B)** volume of NADES, **(C)** volume of ammonia, **(D)** extraction time, **(E)** mass of magnetic particles, **(F)** size of magnetic particles.

The NADES volume significantly influenced extraction recoveries in the ANADES-M-DLLME procedure. The NADES volume was evaluated over a range of 5–40 μL. As shown in Figure 3B, extraction recoveries increased markedly as the NADES volume rose from 5 to 20 μL, then plateaued with further increases. The initial increase is attributed to the greater availability of the NADES phase, which enhances the partitioning of analytes from the aqueous phase. However, beyond 20 μL, the recoveries reached a plateau, likely due to the saturation of analyte extraction from the limited sample volume in the aqueous phase.

Given the alkaline nature of CNS drugs, sample pH governs the charge state of the analytes and directly influences extraction efficiencies. To maintain the molecular (neutral) forms of the analytes for optimal hydrophobic partitioning into the ANADES phase, the volume of ammonia used in preparing alkaline NADES was optimized over the range of 100–600 μL. As evidenced by Figure 3C, the alkaline NADES prepared with 400 μL ammonia (yielding a sample solution pH of 9.49 during extraction; further details are provided in Supplementary Table S2) achieved maximal extraction recoveries.

To ensure analyte partitioning equilibrium during extraction, extraction time was investigated over 4–12 min. As shown in Figure 3D, a 2-min extraction time yielded higher recoveries, with no significant improvement observed at longer times. This rapid equilibrium can be explained by the high surface area of the NADES droplets dispersed in the aqueous phase, which facilitates fast mass transfer of analytes from the aqueous matrix to the extraction phase. Prolonged extraction times did not enhance recoveries and may even lead to analyte loss or back-extraction. Therefore, 2 min was selected as the optimal extraction time.

Effective retrieval of the NADES phase containing target analytes is critically dependent on the mass and size of magnetic particles. To optimize these parameters, systematic investigations were conducted. Magnetic particles (Fe_3_O_4_, 50 nm) with particle mass ranging from 5 to 40 mg were evaluated for their impact on extraction recoveries. As depicted in Figure 3E, the optimal recoveries were achieved with a particle mass of 15 mg. At lower masses, insufficient NADES was captured, leading to incomplete retrieval; further increasing the particle mass beyond 15 mg did not significantly improve the recoveries, indicating that the NADES phase had been fully captured and a plateau was reached. Furthermore, various particle sizes (20 nm, 50 nm, 500 nm) were assessed. Results presented in Figure 3F indicated that 50 nm particles provided high recoveries with superior reproducibility (lower RSD) across all analytes. Therefore, the 50 nm particles were selected for further studies.

Based on the single-factor optimization results, three key parameters significantly influencing the extraction efficiency of CNS drugs were identified: NADES volume, magnetic particle mass, and ammonia volume. To further optimize these three variables, response surface methodology (RSM) was employed, with the mean extraction recoveries of the seven analytes used as the response variable.

#### 3.3.2 Optimization of extraction conditions using Box-Behnken design (BBD)

The optimization was performed using a Box-Behnken design (BBD) as described in Section 2.8. The design comprised 17 experimental runs was employed to investigate three critical variables: NADES volume, magnetic particle mass, and ammonia volume. Each variable was evaluated at three coded levels: −1 (low), 0 (medium), and +1 (high), assessing their impact on the average extraction recoveries of seven CNS drugs. The complete experimental design matrix and corresponding recovery results are provided in Supplementary Tables S3 and S4, respectively.

Data analysis via multiple regression and ANOVA (Supplementary Table S5) confirmed the significance of all model coefficients (*P* < 0.0001), indicating each variable substantially influenced analyte recovery. Lower *P*-values denote greater coefficient significance and stronger correlation between predicted and observed values. Furthermore, the lack-of-fit statistic (*F* = 5.53, *P* = 0.0660) was non-significant at the 95% confidence level, supporting model adequacy.

Model reliability was confirmed by establishing a regression equation relating average extraction recoveries (*Y*) to key parameters: *Y* = 88.76 + 10.26*A* - 5.77*B* - 6.36*C* - 5.40*AC* - 4.30*BC* - 19.08*A* ^2^ - 17.28*C* ^2^ (where *A*: the volume of NADES; *B*: the mass of magnetic particles; *C*: the volume of ammonia).

The model demonstrated high predictive capability, with an *R* ^2^ of 0.9897, an adjusted *R* ^2^ of 0.9765, and a predicted *R* ^2^ of 0.8641, together with adequate precision (23.60) (Ramezani et al., 2017). Variable-response relationships are further visualized in the 3D response surface plots (Figure 4). It should be noted that although the single-factor experiment suggested 15 mg as a suitable mass for magnetic particles, the BBD model, which considers interactive effects among variables, predicted a refined optimum of 12 mg. This value was therefore used in all subsequent validation experiments. Single-factor experiments and multivariate BBD optimization were used to determine the optimal extraction conditions: NADES (HBA:HBD, 1:1), 23 μL; ammonia, 400 μL; extraction time, 2 min; magnetic particles (50 nm) for NADES retrieval, 12 mg. Under these optimized conditions, the mean experimental recoveries for the seven CNS drugs (n = 4) agreed well with model predictions, yielding an accuracy of 97.9% ± 10.5%. This confirmed the model’s predictability and reproducibility.

**FIGURE 4:**
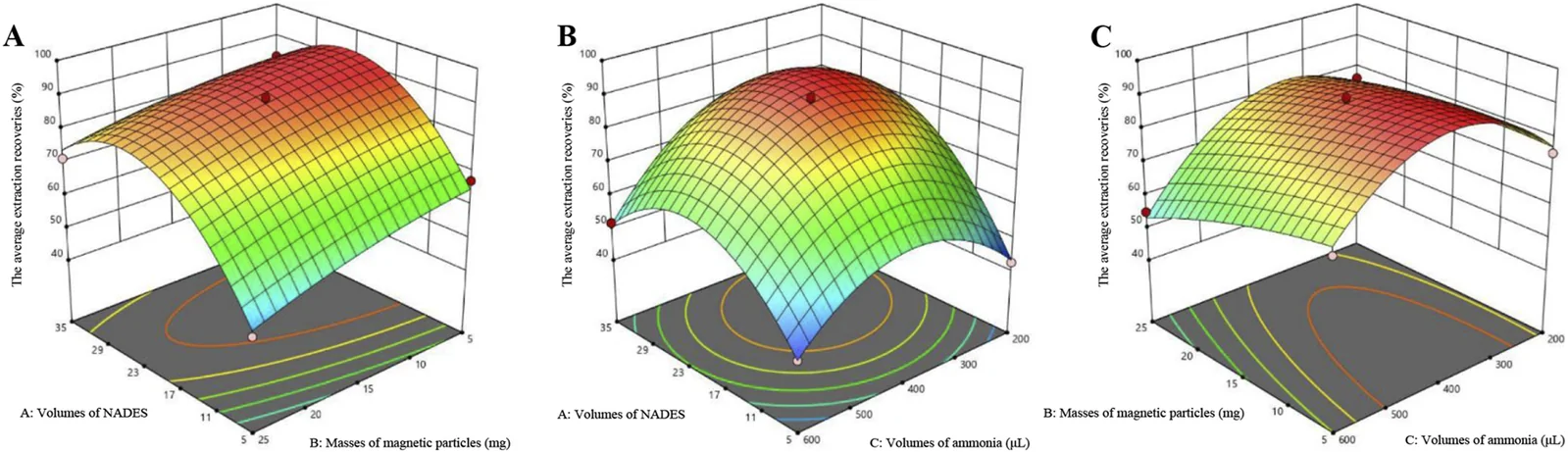
3D response surface plots for the effect of three factors on average extraction recoveries of seven CNS drugs: **(A)** volume of NADES vs. mass of magnetic particles; **(B)** volume of NADES vs. volume of ammonia; **(C)** mass of magnetic particles vs. volume of ammonia.

#### 3.3.3 Optimization of the desorption variables

To ensure complete elution of the target analytes and enhance detection sensitivity, the effects of desorption time and volume of desorption solvent on the extraction recoveries were investigated separately. Following the addition of ethyl acetate for desorption, the aqueous sample solution was vortexed for durations ranging from 0.5 to 5 min. As shown in Supplementary Figure S4A, vortex-mixing for 1.0 min proved sufficient. The influence of desorption solvent volume was evaluated over a range of 0.2–1.0 mL. The results (Supplementary Figure S4B) demonstrated that the maximum enrichment factor was achieved using 200 μL of ethyl acetate.

### 3.4 Evaluation of matrix effects

Matrix effects represent a critical consideration in bioanalytical method development due to their potential impact on quantification accuracy. To systematically evaluate matrix interference for CNS drugs, peak areas from human plasma samples were compared with those from deionized water controls–both spiked at 50 ng/mL, 500 ng/mL, and 5 μg/mL target analyte concentration and processed identically using the developed method. All matrix effect values (Table 1) ranged from 84.9% to 108.0%, demonstrating negligible impact of the plasma matrix on the quantification of CNS drugs. Consequently, standard solutions of CNS drugs were employed for subsequent validation to streamline workflow.

**TABLE 1:** Matrix effects of seven CNS drugs in the plasma samples.

Analyte	Matrix effects (%)		
	50 ng/mL	500 ng/mL	5 μg/mL
LID	91.1	98.8	100.8
AMP	94.0	92.9	102.1
IMP	103.8	105.3	94.7
PMZ	97.2	105.6	93.4
DZP	89.8	104.5	84.9
CMZ	101.6	86.7	106.5
CZP	108.0	102.0	88.2

Matrix effects (%) = A1/A2 × 100% A1: peak area of spiked plasma samples; A2: peak area of neat standard solutions.

### 3.5 Method validation

To validate the proposed ANADES-M-DLLME method under optimized conditions, its analytical characteristics, including linearity, limit of detection (LOD), limit of quantification (LOQ), extraction recoveries, and precision, were investigated. The results are summarized in Table 2. Calibration curves were established using deionized aqueous solutions spiked with seven CNS drugs at varying concentrations and a fixed concentration of internal standards (diphenylamine, DPM; cyclobenzaprine hydrochloride, CBZ) at 50 ng/mL each. Good linearity was observed over the concentration ranges of 5–1000 ng/mL (for LID, AMP, PMZ) and 10–1000 ng/mL (for IMP, DZP, CMZ, CZP), with satisfactory determination coefficients (*R* ^2^) ranging from 0.990 to 0.999. The sensitivity of the method, evaluated by the LOD and LOQ defined at signal-to-noise ratios (S/N) of 3 and 10, respectively, yielded values of 0.61–3.36 ng/mL and 2.05–9.89 ng/mL for the seven CNS drugs. Under optimal conditions, satisfactory extraction recoveries ranging from 85.01% to 99.89% were achieved for all seven CNS drugs. The enrichment factor (EF) values for the seven CNS drugs ranged from 6.65 to 8.79, indicating effective enrichment by the ANADES-M-DLLME method.

**TABLE 2:** Linear ranges, regression data, LODs and LOQs for the determination of the seven CNS drugs.

Analyte	Linear range	Regression line		LOQ	LOD	EF
	(ng/mL)	Linear equation	*R* ^2^	(ng/mL)	(ng/mL)	
LID	5–1000	*Y* = 0.6385*X* – 0.1076	0.9996	2.37	0.73	8.58
AMP	5–1000	*Y* = 0.0921*X* – 0.0529	0.9999	2.05	0.61	7.58
IMP	10–1000	*Y* = 0.3478*X* – 0.2596	0.9984	5.32	1.68	6.66
PMZ	5–1000	*Y* = 0.1051*X* – 0.0532	0.9942	2.11	0.64	7.69
DZP	10–1000	*Y = 0.3048X* – 0.0586	0.9992	5.26	1.49	6.65
CMZ	10–1000	*Y = 0.1342X* – 0.0901	0.9932	5.42	1.74	8.79
CZP	10–1000	*Y = 0.0368X* – 0.0266	0.9907	9.89	3.36	6.72

The precision and accuracy of the method, assessed through intra-day and inter-day relative standard deviations (RSDs) and relative recovery studies, were determined as follows: Intra-day precision and accuracy were evaluated by analyzing spiked aqueous solutions at three concentration levels (50, 100, and 500 ng/mL) with three consecutive injections within a single day. Inter-day precision and accuracy were measured over three consecutive days. As detailed in Supplementary Table S6, the results demonstrate excellent precision (RSDs <13.3%) and accuracy (relative recoveries: 95.1%–114.9%) for quantifying CNS drugs in biofluids, confirming the reliability of the method.

The therapeutic plasma concentrations of the selected CNS drugs were compared with the established linear ranges. According to published TDM guidelines and toxicology references (Hiemke et al., 2018; Schulz et al., 2020), the therapeutic reference ranges are approximately 1.5–5.0 μg/mL for LID, 80–200 ng/mL for AMP, 150–300 ng/mL for IMP, 50–400 ng/mL for PMZ, 100–2000 ng/mL for DZP, 50–300 ng/mL for CMZ, and 350–600 ng/mL for CZP. The linear ranges obtained in this work (5–1000 ng/mL for LID, AMP, and PMZ; 10–1000 ng/mL for IMP, DZP, CMZ, and CZP) cover the therapeutic ranges of AMP, IMP, PMZ, CMZ, and CZP, as well as the lower-to-middle therapeutic range of DZP. For analytes with clinical concentrations above the upper linear limit, such as LID and high-concentration DZP samples, plasma samples can be diluted with blank plasma prior to extraction. The LLOQs (2.05–9.89 ng/mL) are below or close to the lower therapeutic limits, supporting the applicability of the method for TDM of these CNS drugs.

### 3.6 Analysis of biological samples

To assess real-world applicability, plasma samples spiked with seven CNS drugs were analyzed. Due to the unavailability of authentic biological samples from CNS drug-treated patients, validation employed single-blind fortified plasma samples (25, 40, and 80 ng/mL) prepared by spiking blank plasma matrix with analyte stocks. An independent researcher prepared these samples, which were subsequently analyzed by the first author without knowledge of fortification levels until data acquisition. As summarized in Table 3, all seven analytes were successfully detected. Satisfactory relative recoveries ranging from 95.1% to 112.7% were achieved, with all relative standard deviations (RSDs) below 15.1%. These validation metrics confirm the ANADES-M-DLLME method’s suitability for bioanalytical applications.

**TABLE 3:** Determination of the selected drugs in real samples pretreated with ANADES-M-DLLME in single-blind experiences (*n* = 3).

Analytes	25 ng/mL		40 ng/mL		80 ng/mL	
	Recovery (%)	RSD (%)	Recovery (%)	RSD (%)	Recovery (%)	RSD (%)
LID	102.4	4.3	105.2	3.9	95.1	4.5
AMP	100.5	14.8	100.8	11.8	110.5	8.8
IMP	105.0	13.2	110.4	8.4	109.2	6.8
PMZ	112.7	10.5	104.3	10.3	110.5	10.7
DZP	108.6	8.7	103.7	6.9	97.5	8.7
CMZ	105.7	15.1	110.7	5.3	105.5	6.9
CZP	110.5	14.8	102.4	4.8	95.2	7.2

### 3.7 Method greenness evaluation

A current priority in analytical chemistry is the development of analytical workflows incorporating Green Analytical Chemistry (GAC) principles. In accordance with the 12 principles of GAC, several qualitative, semi-quantitative, and quantitative methods have recently emerged to evaluate analytical greenness. The greenness of the proposed methodology was assessed using three established metrics: the Analytical GREEnness Metric (AGREEprep), the complex modified Green Analytical Procedure Index (Complex MoGAPI), and the blue applicability grade index (BAGI).

AGREEprep, an upgraded iteration of the AGREE metric, comprehensively evaluates the greenness of sample preparation procedures against the 10 principles of Green Sample Preparation (GSP) (Wojnowski et al., 2022). This tool converts the ten GSP principles into a 0–1 scoring scale, where the final score reflects holistic compliance. A score of 1.0 indicates optimal green performance across all criteria. As depicted in Figure 5A and Supplementary Table S7, the AGREEprep pictogram yielded a total score of 0.63, significantly above the 0.6 threshold for “green” classification. ComplexMoGAPI integrates the visual strengths of ComplexGAPI with precise quantitative scoring, enabling more accurate and objective analytical method comparisons while providing comprehensive assessment of environmental sustainability (Mansour et al., 2024). The ComplexMoGAPI pictogram in Figure 5B and Supplementary Table S8, characterized by its green outer ring and overall score of 86, demonstrated superior green performance. BAGI, a novel Green Analytical Chemistry (GAC) metric introduced by (Manousi et al., 2023), simultaneously evaluates the practicality and environmental sustainability of analytical methods. This tool employs a scoring system (25–100 scale), color gradients, and pictograms to visualize analytical greenness. Higher BAGI scores indicate superior environmental performance, with Figure 5C and Supplementary Table S9 depicting a score of 67.5 – quantitatively confirming the method’s sustainability.

**FIGURE 5:**
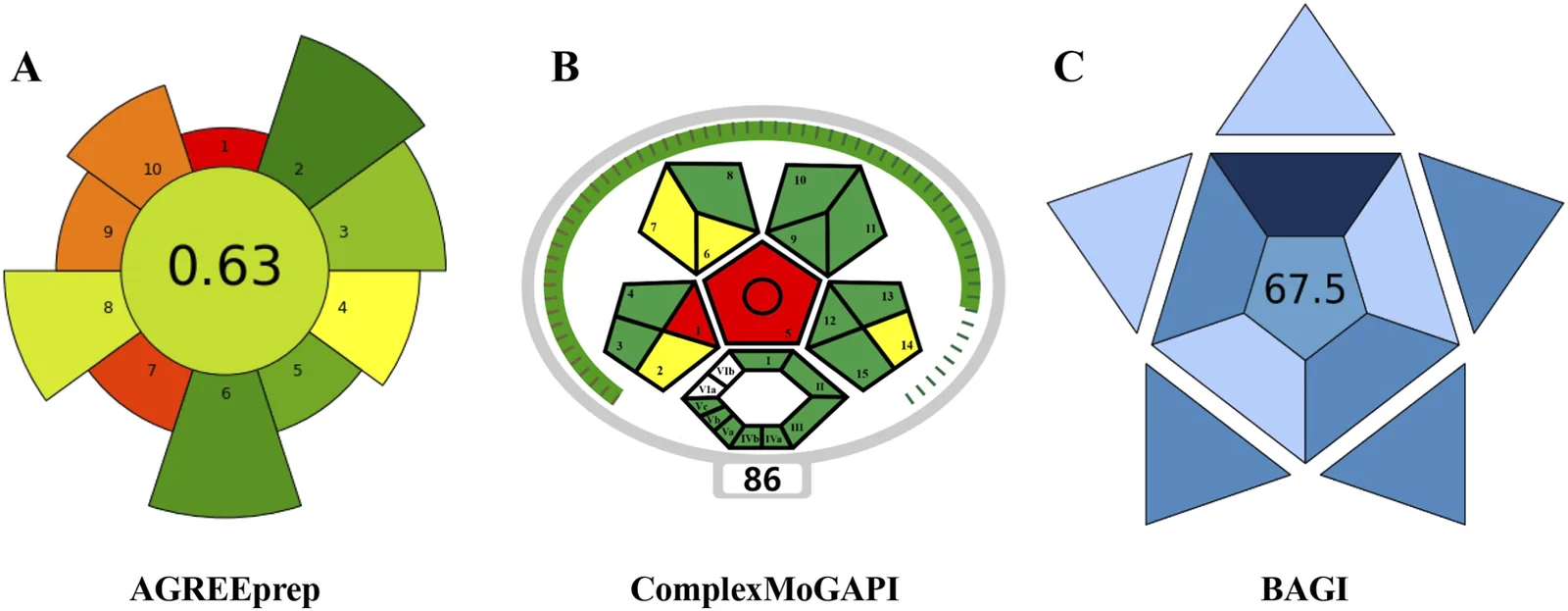
The **(A)** AGREEprep, **(B)** Complex MoGAPI, and **(C)** BAGI assessment results of the green profile of the ANADES-M-DLLME-GC-MS/MS method.

It should be noted that while ethanol (700 µL per sample, used for protein precipitation) and ethyl acetate (200 µL per sample, used for desorption) are significantly greener alternatives to conventional chlorinated solvents, they are volatile organic compounds (VOCs) and still contribute to the overall environmental footprint. However, their consumption per sample is minimal, which mitigates their environmental burden and supports the favorable greenness scores obtained from AGREEprep, Complex MoGAPI, and BAGI. Collectively, the results from the AGREEprep, ComplexMoGAPI, and BAGI metrics validate the developed method’s excellent greenness.

### 3.8 Method advantages

In comparison to previously reported methods also using DLLME for CNS drug determination (Table 4), the proposed approach offers significant practical advantages, particularly in terms of its environmental friendliness, enhanced extraction efficiency, and workflow simplicity.

**TABLE 4:** Comparison of previous CNS drug determination methods using DLLME techniques.

Matrix	Extractant/retrieval strategies of extractant	Mode of pH regulation	Extraction time (min)	Detection	Extraction recoveries	LOD (ng/mL)	References
Plasma	23 μL of NADES (menthol, carvone)/magnetic particle-assisted retrieval	Integrated	∼5	GC- MS/MS	85.1%–99.8%	0.6–3.4	Present work
Urine	Ethyl acetate (500 μL) and chloroform (200 μL)/centrifugation	Manual	>30	LC-MS/MS	14.3%–71.7%	0.25–1.5	Cliville-Cabre et al. (2025)
Whole blood	60 μL of imidazolium-based ionic liquids/centrifugation	Manual	>10	LC-MS/MS	60.0%–100.0%	Not provided	De Boeck et al. (2018)
Plasma	200 μL of chloroform/centrifugation	Manual	>15	UPLC-PDA	93.0%–110.0%	4.0–5.0	Fernández et al. (2016)
Human serum, wastewater	60 μL of dipropylamine/centrifugation	Manual	>35	GC-FID	70.1%–83.5%	0.3–1.0	Behpour et al. (2020)
Human serum, urine	Trifluoroacetic acid (1.0 mL) and 1-dodecanol (200 μL)/solidification	Manual	>10	HPLC-UV	65.0%–78.0%	0.5–1.5	Arghavani-Beydokhti et al. (2023)

A prominent advancement is the utilization of a novel type V NADES, composed of green, non-toxic natural components, as the extractant, avoiding the use of volatile toxic organic solvents. Moreover, the consumption of extractant is markedly lower than in previous reports. Through detailed optimization experiments of NADES composition, the tailored NADES exhibits excellent compatibility with the target analytes. The extraction recoveries obtained by the current method range from 85.1% to 99.8%, significantly surpassing those achieved by other methods.

Furthermore, the DLLME procedure itself is substantially simplified. The alkaline NADES possesses inherent pH regulation capability, eliminating the need for external pH adjustment and thereby reducing operational steps. Moreover, magnetic particle-assisted retrieval of the extractant further enhances operational convenience without compromising efficiency. Based on these optimizations, the extraction time required in this study is significantly shorter than that of the reported methods. In terms of analytical sensitivity, the proposed method, coupled with GC-MS/MS, achieves LODs ranging from 0.61 to 3.36 ng/mL. This sensitivity is comparable to or superior to LODs reported in prior studies (e.g., 0.5–1.5 ng/mL and 4.0–5.0 ng/mL) (Arghavani-Beydokhti et al., 2023; Fernández et al., 2016).

Taken together, these results demonstrate that the proposed ANADES-M-DLLME method offers a simpler, faster, more environmentally friendly, and cost-effective (reduced solvent consumption) approach with high sensitivity for the extraction of CNS drugs from plasma.

## 4 Conclusion

A novel, efficient DLLME method utilizing type V alkaline NADES combined with magnetic particle-assisted extractant retrieval was developed for the determination of central nervous system (CNS) drugs in biological fluids. Unlike conventional NADES-based DLLME methods that require external pH adjustment and centrifugation-based extractant recovery, the proposed ANADES-M-DLLME method integrates pH regulation into the extractant itself and employs magnetic retrieval, thereby eliminating two major operational bottlenecks. This dual innovation—built-in alkalinity and magnetic retrieval—has not been previously reported for NADES-based DLLME. The satisfactory validation parameters confirm that the optimized conditions are robust and applicable for routine analysis. The wide linear ranges and low LODs demonstrate that the method is sensitive enough for therapeutic drug monitoring of CNS drugs in clinical settings. The proposed method offers several distinct advantages, including greenness (non-toxic NADES and minimal solvent consumption), rapidity (short extraction time), cost-effectiveness, and sensitivity for the quantification of CNS drugs. Nevertheless, certain limitations should be acknowledged. First, because the method was validated using spiked plasma samples due to the limited availability of authentic clinical specimens, its applicability to a broader range of analytes or other biological matrices (e.g., urine or cerebrospinal fluid) requires further investigation. Further validation with a larger number of real patient samples will be pursued in future studies. Second, the method relies on GC-MS/MS instrumentation, which is not universally available in routine analytical laboratories and incurs relatively high detection costs. Future work will focus on adapting the developed sample preparation strategy to more accessible and cost-effective analytical instruments. Overall, the proposed method exhibits competitive advantages in terms of being green, cost-effective, and simplified for the accurate and reliable quantification of CNS drugs in biological samples, holding promising potential for clinical therapeutic drug monitoring applications.

## Data Availability

The raw data supporting the conclusions of this article will be made available by the authors, without undue reservation.
