# Supplementary material for: A novel type V natural deep eutectic solvent with built-in pH-regulation for magnetic dispersive liquid-liquid microextraction of central nervous system drugs in plasma samples
Source: Front Chem. 2026 Oct 27;14:1986305. doi: 10.3389/fchem.2026.1986305 (PMC13747954; doi:10.3389/fchem.2026.1986305)
Supplement: Supplementary file 1 [file DataSheet1.docx]

**Fig. S1.** FT-IR spectra of menthol, eucalyptol, carvone and their corresponding NADES at different compositions. Note: NADES 1 was composed of menthol and eucalyptol at a 1:1 molar ratio; NADES 2 was composed of menthol and carvone at a 1:1 molar ratio.

**Fig. S2.** ^1^H NMR characterization of intermolecular interactions. (A): ^1^H NMR spectra of eucalyptol, menthol, and NADES 1 (menthol–eucalyptol); (B): ^1^H NMR spectra of carvone, menthol, and NADES 2 (menthol–carvone). Note: NADES 1 was composed of menthol and eucalyptol at a 1:1 molar ratio; NADES 2 was composed of menthol and carvone at a 1:1 molar ratio.

**Fig. S3.** Comparison of extraction efficiency for seven CNS drugs using the proposed ANADES-M-DLLME method: (A) alkaline NADES 1 as extractant under acidic, neutral, and alkaline conditions, (B) alkaline NADES 2 as extractant under acidic, neutral, and alkaline conditions, (C) NADES 1 and NADES 2 as extractants under alkaline conditions. Note: NADES 1 was composed of menthol and eucalyptol at a 1:1 molar ratio; NADES 2 was composed of menthol and carvone at a 1:1 molar ratio.

**Fig. S4.** Optimization of the desorption variables of the ANADES-M-DLLME method using single-factor experiments: (A) desorption time, (B) volume of desorption solvent.

**Table S1.** The conditions and transitions of MRM for IS and CNS drugs.

| Analyte | Retention time (min) | Precursor ion (*m/z*) | Product ion (*m/z*) | CE (Volt) |
| --- | --- | --- | --- | --- |
| DPM | 9.804 | 169.9 | 66.0 | 35 |
| CBZ | 13.182 | 214.9 | 189.1 | 30 |
| LID | 11.669 | 86.0 | 58.1 | 10 |
|  |  | 86.0 | 42.2 | 45 |
| AMP | 13.287 | 214.9 | 189.1 | 30 |
|  |  | 201.9 | 152.1 | 40 |
| IMP | 13.482 | 233.9 | 218.1 | 30 |
| PMZ | 13.775 | 212.9 | 198.1 | 25 |
|  |  | 179.9 | 152.1 | 30 |
| DZP | 14.700 | 255.8 | 221.1 | 15 |
|  |  | 255.8 | 165.2 | 40 |
| CMZ | 15.102 | 272.0 | 257.0 | 30 |
|  |  | 272.0 | 237.0 | 20 |
| CZP | 18.874 | 325.9 | 242.9 | 15 |
|  |  | 325.9 | 98.8 | 30 |

**Table S2.** The pH of sample solutions regulated by alkaline NADES synthesized with varying volume of ammonia solution.

| Volume of ammonia solution (μL) | pH of sample solutions |
| --- | --- |
| 100 | 9.09 |
| 200 | 9.30 |
| 300 | 9.39 |
| 400 | 9.49 |
| 500 | 9.58 |
| 600 | 9.64 |

**Table S3.**  Coded and real values of chosen variables for Box–Behnken design.

| Variable | Symbol | Range and level | | |
| --- | --- | --- | --- | --- |
|  |  | Low (-1) | Middle (0) | High (+1) |
| Volume of NADES (µL) | A | 5 | 20 | 35 |
| Mass of magnetic particles (mg) | B | 5 | 15 | 25 |
| Volume of ammonia (µL) | C | 200 | 400 | 600 |

**Table S4.**  List of experiments in the Box–Behnken design for the optimization model and average extraction recoveries of seven CNS drugs.

| Experiment number | Factor 1 | Factor 2 | Factor 3 | Response |
| --- | --- | --- | --- | --- |
|  | Volume of NADES (µL) | Mass of magnetic particles (mg) | Volume of ammonia  (µL) | Average extraction recoveries (%) |
| 1 | 20 | 15 | 400 | 90.6 |
| 2 | 20 | 15 | 400 | 90.2 |
| 3 | 20 | 15 | 400 | 88.0 |
| 4 | 20 | 15 | 400 | 87.8 |
| 5 | 20 | 15 | 400 | 87.2 |
| 6 | 35 | 5 | 400 | 83.5 |
| 7 | 20 | 25 | 200 | 76.4 |
| 8 | 20 | 5 | 200 | 75.6 |
| 9 | 35 | 15 | 200 | 74.5 |
| 10 | 35 | 25 | 400 | 71.3 |
| 11 | 20 | 5 | 600 | 70.3 |
| 12 | 5 | 5 | 400 | 67.4 |
| 13 | 20 | 25 | 600 | 55.0 |
| 14 | 35 | 15 | 600 | 51.6 |
| 15 | 5 | 25 | 400 | 47.9 |
| 16 | 5 | 15 | 200 | 42.4 |
| 17 | 5 | 15 | 600 | 41.1 |

**Table S5.**  Analysis of variance (ANOVA) for response surface quadratic model.

| Source | Sum of squares | df | Mean square | *F*-value | *P*-value |
| --- | --- | --- | --- | --- | --- |
| Model | 4646.16 | 9 | 516.24 | 74.73 | < 0.0001 |
| A-Volume of NADES | 842.55 | 1 | 842.55 | 121.97 | < 0.0001 |
| B-Mass of magnetic particles | 266.80 | 1 | 266.80 | 38.62 | 0.0004 |
| C-Volume of ammonia | 323.85 | 1 | 323.85 | 46.88 | 0.0002 |
| *AB* | 13.32 | 1 | 13.32 | 1.93 | 0.2075 |
| *AC* | 116.64 | 1 | 116.64 | 16.89 | 0.0045 |
| *BC* | 64.80 | 1 | 64.80 | 9.38 | 0.0182 |
| *A*^2^ | 1532.83 | 1 | 1532.83 | 221.90 | < 0.0001 |
| *B*^2^ | 19.55 | 1 | 19.55 | 2.83 | 0.1364 |
| *C*^2^ | 1257.26 | 1 | 1257.26 | 182.01 | < 0.0001 |
| Residual | 48.35 | 7 | 6.91 |  |  |
| Lack of Fit | 38.96 | 3 | 12.99 | 5.53 | 0.0660 |
| Pure Error | 9.39 | 4 | 2.35 |  |  |
| Cor Total | 4694.52 | 16 |  |  |  |
| R^2^ | 0.9897 |  |  |  |  |

**Table S6.** Intra- and inter-day relative recoveries and RSD (%) of the developed method at three different spiking levels (50, 100 and 500 ng/mL).

| Analyte | Added (ng/mL) | Intra-day (*n*=3) | | Inter-day (*n*=3) | |
| --- | --- | --- | --- | --- | --- |
|  |  | Recovery (%) | RSD (%) | Recovery (%) | RSD (%) |
| LID | 50 | 101.8 | 3.6 | 104.9 | 3.6 |
|  | 100 | 102.2 | 3.5 | 101.6 | 3.7 |
|  | 500 | 98.1 | 5.5 | 97.7 | 0.9 |
| AMP | 50 | 105.5 | 9.3 | 105.6 | 2.3 |
|  | 100 | 106.7 | 9.7 | 107.0 | 0.6 |
|  | 500 | 112.5 | 9.2 | 110.2 | 1.9 |
| IMP | 50 | 111.0 | 10.2 | 114.5 | 9.7 |
|  | 100 | 113.3 | 6.3 | 113.3 | 2.7 |
|  | 500 | 110.1 | 8.9 | 109.2 | 1.6 |
| PMZ | 50 | 112.6 | 7.4 | 114.2 | 5.7 |
|  | 100 | 105.2 | 13.0 | 108.1 | 8.6 |
|  | 500 | 111.5 | 12.6 | 106.6 | 2.1 |
| DZP | 50 | 105.3 | 3.7 | 104.5 | 5.1 |
|  | 100 | 101.8 | 5.2 | 102.4 | 0.8 |
|  | 500 | 95.9 | 5.6 | 95.1 | 1.1 |
| CMZ | 50 | 101.8 | 9.9 | 108.7 | 12.8 |
|  | 100 | 105.4 | 3.7 | 106.6 | 1.5 |
|  | 500 | 103.5 | 9.3 | 103.3 | 0.3 |
| CZP | 50 | 112.4 | 13.3 | 114.9 | 5.3 |
|  | 100 | 106.8 | 4.5 | 108.7 | 3.8 |
|  | 500 | 96.2 | 8.4 | 96.5 | 1.9 |

**Table S7.** Input parameters used for AGREEprep assessments.

| Criterion | Input | Justification | Weight |
| --- | --- | --- | --- |
| 1. Sample preparation placement | Ex situ | Analytical procedure is ex situ | 1 |
| 2. Hazardous materials | 0.00 g/mL | No hazardous solvents; NADES components and ethanol/ethyl acetate are non-toxic at used volumes | 5 |
| 3. Sustainability, renewability, and reusability of materials | ＞75% of reagents and materials are sustainable or renewable | Menthol, carvone and ethanol are renewable | 4 |
| 4. Waste | ∼2.2 g or mL per sample | Sample (∼2 mL) + ethyl acetate (∼0.2 mL) | 3 |
| 5. Size economy of the sample | 300 μL plasma | Volume of plasma sample used | 2 |
| 6. Sample throughput | ∼60 samples/h | Total extraction + desorption time ∼6 min per batch (10 samples) | 5 |
| 7. Integration and automation | 4 manual steps | (1) Precipitation/centrifugation, (2) extraction, (3) retrieval, (4) desorption; all manual | 2 |
| 8. Energy consumption | ∼100 Wh per sample | Energy requirements for sample preparation | 4 |
| 9. Post-sample preparation configuration for analysis | GC-MS/MS | Triple quadrupole MS detection | 2 |
| 10. Operator's safety | 3 hazard pictograms | Ammonia, ethanol, ethyl acetate | 3 |

**Table S8.** Input parameters used for ComplexMoGAPI assessments.

| Category | Criterion | Input | Score |
| --- | --- | --- | --- |
| SAMPLE PREPARATION | 1 - Collection: | Off-line | Green |
|  | 2 - Preservation: | Chemical or physical | Yellow |
|  | 3 - Transport: | None | Green |
|  | 4 - Storage: | None | Green |
|  | 5 - Type of method: | Extraction required | Red |
|  | 6 - Scale of extraction: | Microextraction | Yellow |
|  | 7 - Solvents/reagents used: | Green solvents/ reagents used | Yellow |
|  | 8 - Additional treatment: | Energy requirements for sample preparation | Green |
| REAGENT AND SOLVENTS | 9 - Amount: | <10 mL (<10 g) | Green |
|  | 10 - Health hazard: | Slightly toxic, slightly irritant; NFPA health hazard score is 0 or 1 | Green |
|  | 11 - Safety hazard: | Highest NFPA flammability, instability score of 0 or 1. No special hazards. | Green |
| INSTRUMENTATION | 12 - Energy: | ≤0.1 kW h per sample | Green |
|  | 13 - Occupational hazard: | Hermetization of the analytical process | Green |
|  | 14 - Waste: | 1–10 mL (1–10 g) | Yellow |
|  | 15 - Waste treatment: | Recycling | Green |
|  | 16 - Quantification: | Yes | Green |
| YIELD AND CONDITIONS | I - Yield: | >89 % | Green |
|  | II - Temperature/time: | Room temperature, <1h | Green |
| RELATION TO GREEN ECONOMY | III - Number of rules met: | 5–6 | Green |
| REAGENTS AND SOLVENTS | IVa - Health hazard: | Slightly toxic, slightly irritant; NFPA health hazard score is 0 or 1 | Green |
|  | IVb - Safety hazard: | Highest NFPA flammability, instability score of 0 or 1. No special hazards | Green |
| INSTRUMENTATION | Va - Technical setup: | Common setup | Green |
|  | Vb - Energy: | ≤0.1 kW h per sample | Green |
|  | Vc - Occupational hazard: | Hermetization of the analytical process | Green |

**Table S9.** Input parameters used for BAGI assessments.

| Criterion | Input | Score |
| --- | --- | --- |
| 1. Type of analysis | Quantitative and confirmatory | Dark blue (10 points) |
| 2. Multi- or single-element analysis | Multi-element analysis for 6–15 compounds of the same chemical class or 2–15 compounds of different chemical classes | Blue (7.5 points) |
| 3. Analytical technique | Sophisticated instrumentation (e.g., LC-MS, GC-MS, ICP-MS, homemade interfaces, homemade automatic systems) | Light blue (5 points) |
| 4. Simultaneous sample preparation | 13–95 | Blue (7.5 points) |
| 5. Sample preparation | Miniaturized extraction sample preparation (e.g., SPME, DLLME, MEPS, SBSE, d-SPE, FPSE) | Light blue (5 points) |
| 6. Samples per h | 2–4 | Light blue (5 points) |
| 7. Reagents and materials | Need to be synthesized in the lab with common instrumentation and in a simple way. | Light blue (5 points) |
| 8. Preconcentration | Preconcentration is required. Required sensitivity is met with one-step preconcentration. | Blue (7.5 points) |
| 9. Degree of automation | Semi-automated with common devices (HPLC autosampler, etc.) | Blue (7.5 points) |
| 10. Amount of sample | 101–500 μL (or mg) bioanalytical samples 10.1–50 mL (or g) food/ environmental. | Blue (7.5 points) |
